# Acute Treatment with Docosahexaenoic Acid Complexed to Albumin Reduces Injury after a Permanent Focal Cerebral Ischemia in Rats

**DOI:** 10.1371/journal.pone.0077237

**Published:** 2013-10-23

**Authors:** Tiffany N. Eady, Larissa Khoutorova, Daniela V. Anzola, Sung-Ha Hong, Andre Obenaus, Alena Mohd-Yusof, Nicolas G. Bazan, Ludmila Belayev

**Affiliations:** 1 Neuroscience Center of Excellence, School of Medicine, Louisiana State University Health Sciences Center, New Orleans, Louisiana, United States of America; 2 Department of Neurosurgery, School of Medicine, Louisiana State University Health Sciences Center, New Orleans, Louisiana, United States of America; 3 Department of Pediatrics, School of Medicine, Loma Linda University, Loma Linda, California, United States of America; National Insttitute on Drug Abuse, United States of America

## Abstract

Docosahexaenoic acid complexed to albumin (DHA-Alb) is highly neuroprotective after temporary middle cerebral artery occlusion (MCAo), but whether a similar effect occurs in permanent MCAo is unknown. Male Sprague-Dawley rats (270–330 g) underwent permanent MCAo. Neurological function was evaluated on days 1, 2 and 3 after MCAo. We studied six groups: DHA (5 mg/kg), Alb (0.63 or 1.25 g/kg), DHA-Alb (5 mg/kg+0.63 g/kg or 5 mg/kg+1.25 g/kg) or saline. Treatment was administered i.v. at 3 h after onset of stroke (n = 7–10 per group). *Ex vivo* imaging of brains and histopathology were conducted on day 3. Saline- and Alb-treated rats developed severe neurological deficits but were not significantly different from one another. In contrast, rats treated with low and moderate doses of DHA-Alb showed improved neurological score compared to corresponding Alb groups on days 2 and 3. Total, cortical and subcortical lesion volumes computed from T2 weighted images were reduced following a moderate dose of DHA-Alb (1.25 g/kg) by 25%, 22%, 34%, respectively, compared to the Alb group. The total corrected, cortical and subcortical infarct volumes were reduced by low (by 36–40%) and moderate doses (by 34–42%) of DHA-Alb treatment compared to the Alb groups. In conclusion, DHA-Alb therapy is highly neuroprotective in permanent MCAo in rats. This treatment can provide the basis for future therapeutics for patients suffering from ischemic stroke.

## Introduction

Ischemic stroke is one of the leading causes of morbidity and mortality worldwide. Tissue plasminogen activator, tPA, is currently the only therapeutic option available for stroke treatment. Its thrombolytic properties provide a means to break up blood clots during the hyper-acute phase of stroke, allowing for reperfusion of the ischemic area. Unfortunately, the use of tPA is restricted by a narrow therapeutic window (it is only approved to be administered up to 4.5 h after stroke onset) and has multiple absolute contraindications, especially for individuals with any increased bleeding risk. As a result, only 3%–5% of patients qualify for this therapy. In pre-clinical studies, the neuroprotective potential of new therapies is assessed by several variations of experimentally-induced ischemia but these tend to be transient focal cerebral ischemia models. Because the majority of strokes do not resolve with immediate reperfusion, it is vital that potential therapeutic compounds are studied using a permanent occlusion animal model as well.

This study was prompted by our earlier findings demonstrating that omega-3 essential fatty acids (found in fish oil) and human serum albumin (Alb) are beneficial in ameliorating cerebral ischemic injury in rodent stroke models [1], [2]. Docosahexaenoic acid (DHA; 22∶6, n-3), an omega-3 essential fatty acid provided in the diet, is concentrated and avidly retained in membrane phospholipids of the central nervous system. Recently, we have shown that DHA therapy at low and medium doses improves outcomes following focal cerebral ischemia [3]. DHA administration results in neurobehavioral recovery, reduces brain infarction and brain edema, activates DHA-derived neuroprotectin D1 (NPD1) synthesis in the penumbra, and promotes cell survival when administered up to 5 h after focal cerebral ischemia in rats [1], [4].

Preclinical studies have established that administration of Alb at high doses decreases infarct volume, reduces brain swelling [5], [6] and improves local cerebral perfusion in affected tissue [7] when administered up to 4 h after focal cerebral ischemia in rats [2]. However, administration of high-dose Alb expands intravascular volume which may lead to pulmonary edema and congestive heart failure in patients [8]. We suspected that if DHA were complexed with Alb, it might be possible to achieve neuroprotection at lower, more clinically achievable doses. Recently, we reported that the DHA-Alb complex affords high-grade neuroprotection in transient focal cerebral ischemia at moderate doses [9], [10], but whether a similar effect occurs in permanent MCAo is unknown.

## Materials and Methods

### Animal Preparation

The present study was approved by the Institutional Animal Care and Use Committee of the Louisiana State University Health Sciences Center, New Orleans. Male Sprague-Dawley rats (Charles River Lab., Wilmington, MA) weighing 279–340 g were fasted overnight but allowed free access to water. Anesthesia was induced with 3% isoflurane in a mixture of 70% nitrous oxide and 30% oxygen. All rats were orally intubated, mechanically ventilated, and femoral arterial and venous catheters were inserted for blood sampling and drug infusion. Body and cranial (left temporalis muscle) temperatures were monitored and held at normothermic levels (36–37°C) during surgical procedures. Arterial blood gases, pH, plasma glucose, hematocrit, and mean arterial blood pressure were measured 15 min before, 15 min after MCAo and 15 min after treatment (3 h 15 min after onset of MCAo).

### Middle Cerebral Artery occlusion (MCAo)

The right middle cerebral artery (MCA) was permanently occluded by an intraluminal filament, as we previously described [11]. In brief, the right common carotid artery (CCA) was exposed through a midline neck incision and dissected free of the surrounding nerves. The occipital branches of the external carotid artery (ECA) were coagulated, and the pterygopalatine artery was ligated. A 4-cm length of 3-0 monofilament nylon suture coated with poly-L-lysine was inserted via the proximal ECA into the internal carotid artery and MCA until mild resistance was felt. Following suture placement, the neck incision was closed. Animals then were allowed to awaken from anesthesia and were tested using a standardized neurobehavioral battery. The animals were allowed to survive for 3 days with free access to food and water.

### Behavioral Tests

Behavioral tests were performed by an observer blinded to the treatment groups at 60 min after MCAo onset and then on days 1, 2 and 3. The battery consisted of two tests that have been used previously to evaluate various aspects of neurologic function: (1) the postural reflex test to examine upper body posture while the animal is suspended by the tail, and (2) the forelimb placing test to examine sensorimotor integration in forelimb placing responses to visual, tactile and proprioceptive stimuli. Neurological function was graded on a scale of 0–12 (normal score = 0, maximal score = 12), as previously described [11]. Only those animals with a high-grade neurological deficit (10 or greater) were used. In our experience, virtually every rat has a neurological deficit of at least 10 at 60 min after MCAo onset.

### Preparation of DHA–Alb Complex

Docosahexaenoic acid in acid form (Cayman Chemical, Ann Arbor, MI) was physically complexed to human albumin by incubating 20 ml of human serum albumin (25%; Baxter, Westlake Village, CA) with 5 mg DHA/g albumin (molar ratio = 0.2) in a shaking incubator at 4°C for 30 minutes with vortex mixing every 5 minutes, as we previously described [9]. Aliquots were extracted, and free fatty acids (FFA) were isolated by thin-layer chromatography, derivatized to fatty acid methyl esters, and analyzed by gas liquid chromatography. Each vial was aliquoted in 5-mL samples and kept under nitrogen in a cold room; vials were gassed with nitrogen every week. The DHA–Alb complex contained 2.1±0.1 µmol DHA per milliliter of albumin and was stable for at least 2 months.

### Experimental Groups

Animals were randomly assigned to six treatment groups: DHA (5 mg/kg), Alb (0.63 or 1.25 g/kg), DHA-Alb (5 mg/kg+0.63 g/kg or 5 mg/kg+1.25 g/kg) or saline. Treatment was administered i.v. 3 h after stroke onset (n = 7–10 per group) by an investigator blinded to the experimental groups.

### *Ex vivo* MRI Data Collection and Analysis

High resolution e*x vivo* magnetic resonance imaging (MRI) data were acquired from 4% paraformaldehyde fixed brains (day 3) using an 11.7 T Bruker Advance 8.9 cm horizontal bore instrument equipped with a 89 mm (ID) receiver coil (Bruker Biospin, Billerica, MA). High-resolution T2 weighted images (T2WI) were acquired with the following parameters: repetition time/echo time (TR/TE) = 3278.3/20 msec, 256×256 matrix, 0.75 mm slice thickness, 0.75 mm interleaved, with 2 averages, number of slices = 25; and field of view (FOV) = 2.0 cm for a total imaging time of 28 min. We have previously reported no volumetric differences between *in vivo* and *ex vivo* MRI infarct assessment [12]. Volumetric analyses of the total brain volume and percent lesion/brain volume were performed on T2WI using Cheshire image processing software (Hayden Image Processing Group, Waltham, MA) and Amira (Mercury Computer Systems, Visage Imaging, Inc., San Diego, CA). T2WI-derived lesion volumes were measured for all cortical and subcortical brain regions.

### Histopathology

After completion of *ex vivo* MRI studies, ten-micron-thick sections were cut in the coronal plane and stained with thionine (Nissl). Sections were digitized at nine standardized coronal levels and cortical and subcortical infarct areas were measured and analyzed using MCID™ Core imaging software (Linton, Cambridge, UK) as previously described [11]. An investigator blinded to the experimental groups outlined the zones of infarction (which were clearly demarcated) as well as the left and right hemispheres of each section. Infarct volume was calculated as the integrated product of cross-sectional area and intersection distance and corrected for brain swelling.

### Statistical Analysis

Data are presented as mean values ± SEM. Repeated measure analysis of variance (ANOVA) followed by Bonferroni procedures were used for multiple comparisons. Two-tailed Student’s *t* tests were used for two-group comparisons. Differences at *P*<0.05 were considered statistically significant.

## Results

### Physiological Variables

Rectal and cranial temperatures, blood pressure, plasma glucose and blood gases in all animals of this study showed no significant differences between groups (Table 1). Alb and DHA-Alb therapy led to the expected moderate reduction in hematocrit compared to the vehicle groups (Table 1). There were no adverse side effects observed after treatment in all groups.

**Table 1:** Physiological variables.

	Saline	DHA	Alb	DHA-Alb	Alb	DHA-Alb
		5 mg/kg	0.63 g/kg	0.63 g/kg	1.25 g/kg	1.25 g/kg
	(n = 10)	(n = 7)	(n = 10)	(n = 10)	(n = 10)	(n = 10)
***15 min before MCAo***						
Rectal temperature (°C)	37.3±0.06	37.3±0.14	37.3±0.06	37.4±0.15	36.9±0.15	36.9±0.07
Cranial temperature (°C)	36.9±0.14	36.9±0.19	36.9±0.11	37.0±0.15	37.0±0.09	36.9±0.14
pH	7.46±0.01	7.45±0.01	7.48±0.01	7.46±0.02	7.46±0.01	7.45±0.01
PO2, mm Hg	110±8	100±4	111±7	124±7	113±5	100±7
PCO2, mm Hg	38±0.6	40±0.9	38±0.6	38±0.3	39±0.7	45±6.1
Plasma glucose, mg/dL	173±7	183±9	177±13	154±5	183±10	184±8
Hematocrit, %	45±1.0	44±0.8	44±0.5	44±0.7	45±0.6	45±0.6
MABP	114±3	113±7	107±5	107±4	109±4	132±29
Body weight (g)	327±9	334±9	310±8	327±5	352±9	325±2
***15 min after MCAo***						
Rectal temperature (°C)	37.5±0.10	37.5±0.16	37.5±0.12	37.3±0.10	36.9±0.09	37.0±0.11
Cranial temperature (°C)	37.1±0.10	36.9±0.15	37.1±0.15	36.8±0.11	36.7±0.08	36.9±0.12
pH	7.45±0.01	7.46±0.01	7.47±0.01	7.44±0.02	7.44±0.01	7.44±0.01
PO2, mm Hg	101±6	94±4	96±3	99±2	109±6	97±7
PCO2, mm Hg	40±0.6	40±0.7	38±0.6	41±0.6	40±0.6	45±6.1
Plasma glucose, mg/dL	177±9	168±5	169±10	156±7	176±8	186±8
Hematocrit, %	45±1.1	46±0.7	45±0.7	45±1.0	46±0.6	44±0.7
MABP	132±3	139±7	124±5	133±7	122±5	151±30
***3*** ***h 15 min after treatment***						
Rectal temperature (°C)	37.8±0.17	37.9±0.12	37.6±0.11	37.4±0.07	37.4±0.19	37.5±0.18
Cranial temperature (°C)	37.1±0.17	37.5±0.11	37.5±0.24	37.8±0.11	37.1±0.16	37.2±0.16
Hematocrit, %	44±1.0	43±1.0	39±0.8*	38±0.5*	37±0.4*	36±0.4*
MABP	109±4	115±4	97±2	106±4	98±4	105±3
***1 day after treatment***						
Rectal temperature (°C)	38.6±0.18	38.5±0.18	38.2±0.13	38.6±0.12	38.8±0.10	38.9±0.19
Body weight (g)	293±9	293±10	273±7	285±4	309±7	289±2
***2 days after treatment***						
Rectal temperature (°C)	38.2±0.24	37.4±0.23	37.6±0.15	37.8±0.19	38.0±0.11	38.0±0.12
Body weight (g)	271±12	273±13	250±7	261±3	282±7	264±4
***3 days after treatment***						
Rectal temperature (°C)	36.9±0.36	36.5±0.66	35.6±1.04	36.7±0.15	36.1±0.45	36.9±0.23
Body weight (g)	256±13	262±13	234±7	247±3	265±6	252±7

Values are mean ± SEM.

MCAo, middle cerebral artery occlusion.

MABP, mean arterial blood pressure.

* different from saline group (p<0.05, Student’s t-test).

### Neurobehavioral Assessment

**Figure 1:**
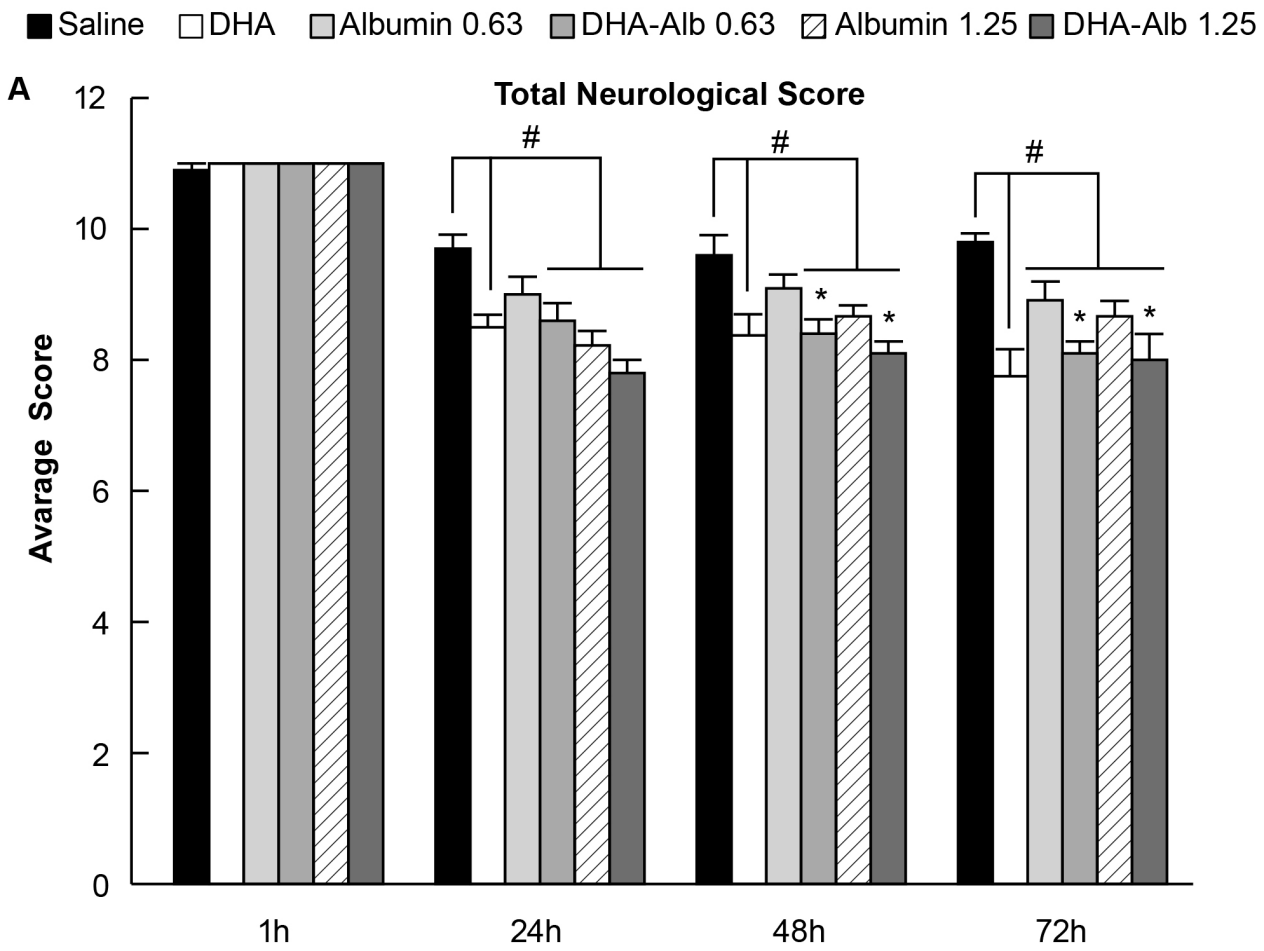
Total neurological score (normal score = 0, maximal score = 12) during MCAo (60 min) and on days 1, 2 and 3 after treatment. Treatment with DHA-Alb significantly improved neurological scores at 48 and 72 h. Values shown are means ± SEM. ^#^ *P*<0.05 versus saline group; *P<0.05 versus Alb (0.63 or 1.25 g/kg) groups (two-way repeated-measures ANOVA).

Prior to middle cerebral artery occlusion, neurological score was normal (score = 0) in all animals. High-grade behavioral deficits (score = 10–11) were present in all animals when tested at 60 min after MCAo (Fig. 1A); thus, no animals required exclusion on the basis of an inadequate degree of cerebral ischemia. Saline (n = 10)- and Alb-treated rats (n = 10 in both treated groups) developed severe neurological deficits and showed no differences in behavioral deficits throughout the 3-day survival period. In contrast, rats treated with low and moderate doses of DHA-Alb (n = 10 in both groups) showed improved neurological scores compared to Alb on days 2 and 3 (Fig. 1A).

### T2WI-derived Lesion Volumes

**Figure 2:**
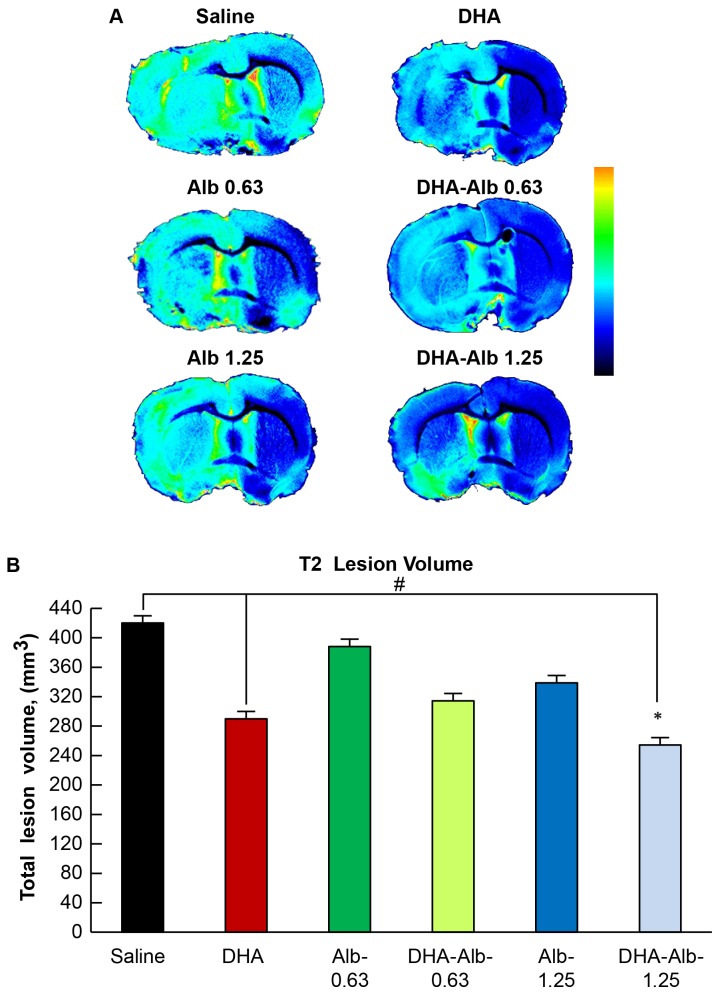
Representative *ex vivo* T2WI from all groups on day 3 after MCAo. Panel A: T2WI were pseudocolored to identify regions of abnormality (green to red) compared to normal brain tissues (black to blue). T2 hyperintensities were observed in the cortex and striatum of saline- and Alb-treated rats, consistent with edema formation. In contrast, DHA-Alb- (1.25 g/kg) treated animals had smaller lesion size, with mostly subcortical involvement. Panel B: Total lesion volumes were computed from T2WI. Lesion volume was reduced in DHA-Alb (1.25 g/kg) and DHA groups compared to the saline group. Treatment with DHA-Alb (1.25 g/kg) significantly reduced total lesion volumes compared to the corresponding Alb-treated group. Values shown are means ± SEM. ^#^ *P*<0.05 versus saline group; *P<0.05 versus Alb (1.25 g/kg) group (two-way repeated-measures ANOVA).

T2WI revealed large lesions involving cortical and subcortical areas of the brain in saline (n = 9)- and Alb-treated animals (Fig. 2A). Rats treated with DHA (n = 7) and DHA-Alb (0.63 g/kg; n = 10) had smaller lesion volumes in the cortex and subcortical areas. In contrast, a moderate dose of the DHA-Alb (1.25 g/kg; n = 8) reduced lesion volume, which was mostly localized to the subcortex and a small portion of the cortex (Fig. 2A). Cortical and subcortical lesion volumes computed from T2WI images were reduced by a moderate dose of DHA-Alb compared to Alb-treated group (cortex: 202±26 vs. 259±9 mm^3^ and subcortex: 52±9 vs. 80±6 mm^3^, respectively). Total lesion volume, computed from T2WI was significantly reduced by treatment with DHA-Alb (1.25 g/kg) compared to the corresponding Alb-treated group (Fig. 2B). DHA treatment reduced total lesion volume compared to the saline group (Fig. 2B).

### Histopathology

**Figure 3:**
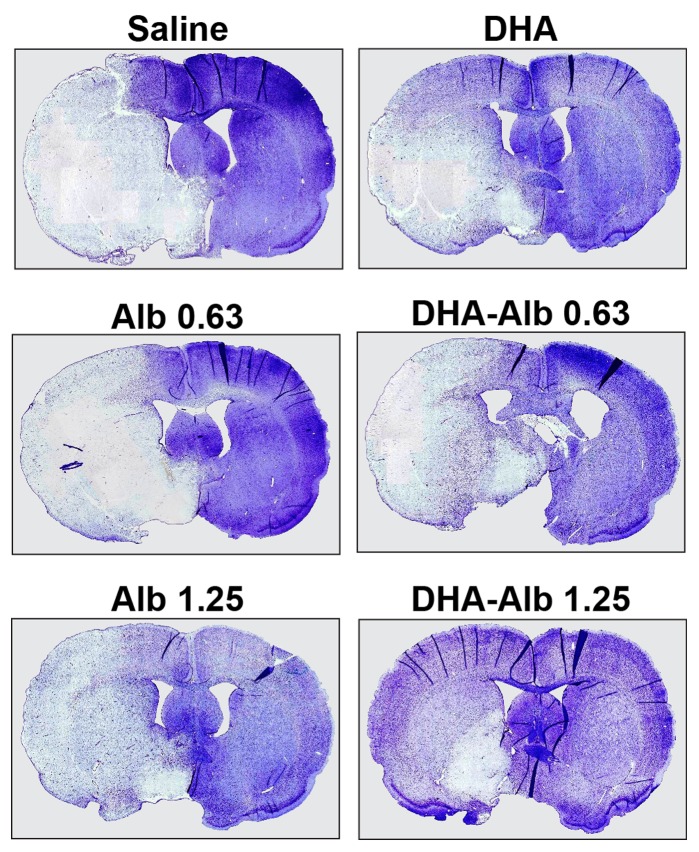
Computer-generated MosaiX-processed images of Nissl stained paraffin-embedded brain sections from rats treated with saline, DHA, Alb (0.63 and 1.25 g/kg) and DHA-Alb (0.63 and 1.25 g/kg) on day 3 after stroke. Saline- and both Alb-treated animals show large cortical and subcortical infarction. DHA-Alb- (0.63 g/kg) and DHA-treated rats showed moderate infarct involving cortical and subcortical regions. In contrast, rats treated with DHA-Alb (1.25 g/kg) showed less extensive damage, mostly in the subcortical area.

**Figure 4:**
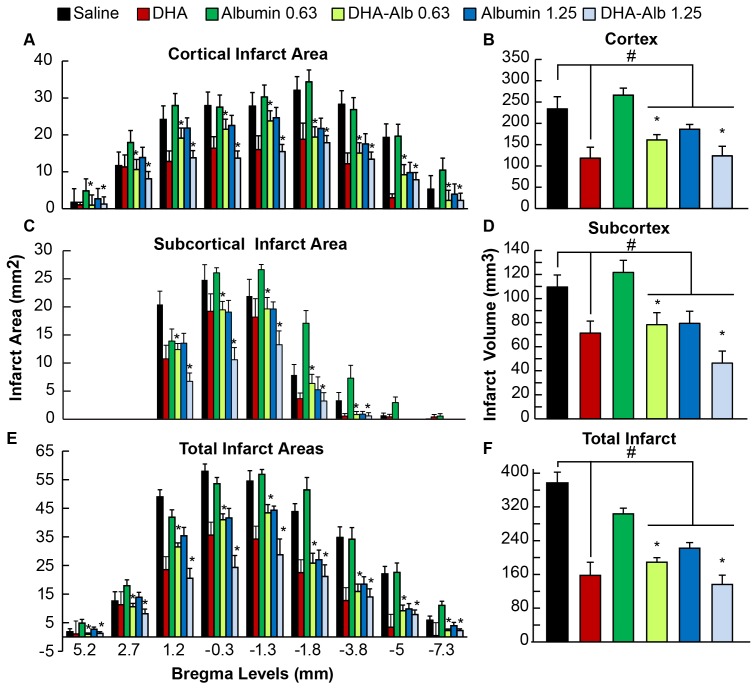
Histopathology on day 3 of survival. Cortical, subcortical and total integrated infarct areas and volumes in rats after permanent MCAo. Treatment with both doses of DHA-Alb reduced cortical, subcortical and total infarct volumes when treatment was administered at 3 h after onset of MCAo. Data are mean ± SEM. ^#^ *P*<0.05 versus saline group; *P<0.05 versus Alb (0.63 or 1.25 g/kg) groups (repeated-measures ANOVA followed by Bonferroni tests).

Brains of saline (n = 9) and both Alb-treated rats (n = 10 in each group) exhibited a consistent pannecrotic lesion involving both cortical and subcortical (mainly striatal) regions of the right hemisphere, characterized microscopically by destruction of neuronal, glial, and vascular elements (Fig. 3). In contrast, infarct size was reduced in rats treated with a moderate dose of DHA-Alb (Fig. 3). Cortical, subcortical and total infarct areas were significantly smaller in both DHA-Alb groups (n = 10 in both groups) compared to saline and corresponding Alb groups (Figs. 4A, C, E). The total corrected infarct, cortical and subcortical infarct volumes were reduced by lower (by 36–40%) and moderate doses (by 34–42%) of DHA-Alb compared to the Alb groups (Figs. 4B, D, F). Six animals died during the experiment: two rats in the saline group (died on days 1 and 2), one rat in the DHA group (died on day 1), two rats in the Alb (0.63 g/kg) group (died on days 1 and 2) and one rat in the Alb (1.25 g/kg) group (died on day 1). DHA-Alb therapy led to 100% survival rate after permanent MCAo. No animals died in any DHA-Alb treated groups.

## Discussion

The goal of our study was to determine whether acute DHA-Alb administration was efficacious in protecting the brain after a permanent focal ischemic insult. Our results clearly indicate that DHA-Alb therapy substantially improves neurological function and reduces T2WI lesion and infarct volumes in rats after permanent MCAo.

Recent research has shown that omega-3 fatty acids found in fish may reduce risk of stroke and coronary heart disease [13]. Docosahexaenoic acid (DHA; 22∶6n-3), a member of omega-3 essential fatty acid family, is a major component of brain membrane phospholipids [14]. It is necessary for ion channels, receptors, and transporters to maintain their proper physical conformation and is involved in memory, synaptic membrane biogenesis and functions, and neuroprotection [14]. After ischemic stroke, free DHA is released from cell membranes and enzymatically converted into a variety of bioactive lipid mediators, many of which have neuroprotective activity. After large or multiple brain injuries, decreases in the stores of omega-3 polyunsaturated fatty acids may potentially result in clinically significant deficits in the brain’s capacity to prevent and recover from injury. Depletion of brain DHA results in extensive loss of sensory, behavioral, and cognitive function both in animals and humans [15]. Recently, we have demonstrated that DHA therapy at low and medium doses improves behavioral outcomes and reduces brain infarction following 2 h of transient MCAo [3]. DHA administration facilitated neurobehavioral recovery and histological protection even when treatment was delayed by up to 5 h post-insult [1]. In addition to DHA, some novel DHA derivatives, such as neuroprotectin D1 (NPD1), have been associated with reduced inflammation and activation of antiapoptotic pathways, two main mechanisms of injury implicated in ischemic stroke [16], [17].

Our previous studies show that high doses of human serum albumin are highly neuroprotective when administered intravenously within a therapeutic window extending up to 4 h after onset of MCAo [2]. Treatment with albumin improves behavior, reduces brain infarction and swelling [5], [6], improves local cerebral blood flow [7] and reduces blood-brain barrier permeability [18]. On the other hand, administration of high-dose Alb causes expansion of intravascular volume and may lead to pulmonary edema and congestive heart failure in certain high risk patients [8].

We predicted that if DHA were complexed with Alb, it might be possible to achieve neuroprotection at lower, more clinically-feasible doses and extend the therapeutic window after experimental stroke. Recently, we reported that the DHA-Alb complexed at moderate doses (0.63 and 1.25 g/kg) affords high-grade neuroprotection in transient focal cerebral ischemia, equaling or exceeding that afforded by native Alb or DHA alone [9]. Thus, we used these doses in the present study of permanent focal cerebral ischemia. Administration of DHA-Alb extended the therapeutic window to 7 h after stroke onset [10]. In addition, DHA-Alb treatment decreased ED-1-positive microglia cells in the penumbra and increased NeuN positive neurons, GFAP positive astrocytes and SMI-71 positive vessels in the ischemic penumbra and core [10]. To date, no study has examined the effect of DHA-Alb on permanent MCAo. Our current study demonstrated that the DHA-Alb complex afforded high-grade neuroprotection, equaling or exceeding that provided by native albumin, and produces no observable adverse effects in young-adult experimental animals.

Omega-3 fatty acids are highly concentrated in the brain and appear to be important for cognitive and behavioral function. Recent studies reported improvements in cognitive functions following DHA or dietary omega-3 fatty acid supplementation in elderly people with age-related cognitive decline and also reported a lowered risk of stroke and incidents of dementia [19], [20]. Animal studies demonstrated that fish oil treatment was able to improve behavioral function and reduce brain infarction in rats subjected to global [21] and focal cerebral ischemia [1]. In our study, administration of two different doses of DHA-Alb at 3 h after the onset of stroke improved the behavioral score when compared to corresponding Alb-treated groups. Neurobehavioral improvement was detected at 48 h after onset of stroke and exceeded that of native Alb throughout the 3-day survival period.

This is the first report demonstrating neuroprotection by DHA-Alb in the setting of permanent MCAo. Models resulting in permanent ischemia mimic clinical stroke without reperfusion. However, because of greater mortality with permanent occlusion models, most preclinical studies use transient MCAo. During preclinical investigations, it is vital that the potential for new therapies to ameliorate the consequences of clinically significant permanent occlusions are assessed in an animal model. Two doses of Alb (1.25 and 2.5 g/kg) were studied previously in the setting of permanent MCAo [22]. Only higher-dose Alb therapy (2.5 g/kg) significantly improved the neurological score and reduced total infarct volume (by 32%) at 24 h compared to vehicle rats, when administered at 2 h after onset of stroke. However, administration of high-dose Alb may lead to development of pulmonary edema in 13% of patients [8]. We suspected that if DHA were complexed with Alb, it might be possible to achieve neuroprotection at lower, more clinically achievable doses. In the current protocol, we used moderate doses of DHA-Alb, administered treatment at 3 h and extended survival period to 3 days after stroke. Our study demonstrates that a moderate dose of DHA-Alb (1.25 g/kg) reduced total, cortical and subcortical lesion volumes computed from T2WI images by 25%, 22%, and 34%, respectively, compared to the Alb groups at 3 days after stroke. In addition, histological evaluation of the brains confirmed that total corrected, cortical and subcortical infarct volumes were reduced by lower (by 36–40%) and moderate doses (by 34–42%) of DHA-Alb treatment when compared to the corresponding Alb groups.

The mechanism by which DHA-Alb provides neuroprotection is not completely understood. Previous studies have established that Alb is actively involved in plasma transport of FFA [23]. Because transient MCAo triggers a massive loss of phospholipid-acyl groups [24], the systemic supply of FFA to the brain, mainly arachidonic acid (AA) and DHA, may be essential to support the repair of neuronal membranes [25]. We recently demonstrated that Alb contributes to the systemic mobilization of all FFA, including the essential fatty acids linoleic acid (18∶2n-6) and linolenic acid (18∶3n-3), precursors of AA and DHA, respectively [26]. In addition, FFA carried by Alb may also be used as an alternative source of energy. In brain regions susceptible to transient cerebral ischemic damage, the mitochondrial generation of ATP is impaired due to a reperfusion-dependent sustained reduction of pyruvate dehydrogenase (PDH) activity [27]. The neuronal beta-oxidation of FFA supplied by Alb may be an alternative source of acetyl-CoA, thus bypassing the deficient PDH pathway. This would contribute to restoring oxidative energy metabolism and the efficient ATP production necessary for the rapid recovery from a cerebral ischemic insult. Finally, MCAo selectively activates the mobilization of omega-3 polyunsaturated fatty acids by Alb, thus supporting the neuronal demands of DHA to repair and rebuild synaptic connections damaged by ischemic insult [26].

In summary, this is the first report demonstrating the neuroprotection of DHA-Alb on permanent focal cerebral ischemia. The present data shows that treatment with DHA-Alb complex results in high-grade neuroprotection, equaling or exceeding that afforded by native albumin, and produced no observable adverse effects in young-adult experimental animals. It improves behavioral scores and reduces total lesion volumes computed from T2WI and infarct volumes, even when treatment is delayed up to 3 h after onset of stroke. These results are encouraging and provide an additional rationale to move forward with clinical trials to investigate the efficacy of DHA-Alb in the treatment of acute ischemic stroke.
